# Enhancing dataset discovery and interoperability, and mobilizing the community via challenges

**DOI:** 10.1002/alz70859_101426

**Published:** 2025-12-25

**Authors:** Rebecca Li, Julie Wood

**Affiliations:** ^1^ Vivli, Boston, MA USA

## Abstract

**Background:**

Alzheimer's disease (AD) research increasingly relies on the ability to access and analyze diverse datasets to advance scientific understanding and therapeutic development. Vivli, a leading global data‐sharing platform, facilitates data discovery and interoperability by providing researchers with access to a vast repository of clinical trial data across multiple therapeutic areas. Currently, Vivli hosts 88 individual participant level datasets related to Alzheimer's disease, offering an unprecedented opportunity to drive innovation through secondary data analyses. Leveraging its experience in conducting data challenges across various fields, Vivli is prepared to collaborate with the Alzheimer’s Disease Data Initiative to launch a new challenge aimed at accelerating discoveries in AD research.

**Method:**

Vivli employs a comprehensive data‐sharing ecosystem that promotes interoperability through FAIR principles. Through partnerships with industry, academia, and non‐profit organizations, Vivli makes high‐quality datasets available and enables researcher access to raw data via a secure and user‐friendly platform. The proposed AD data challenge, in collaboration with the AD Data Initiative, will focus on leveraging Vivli’s interoperable datasets to address critical research questions and engage a global research community.

**Result:**

To date, Vivli has successfully conducted numerous data challenges that have resulted in actionable insights and novel findings across various therapeutic areas. These challenges have fostered cross‐disciplinary collaboration and innovation, demonstrating the value of secondary data analyses. In Alzheimer's disease, Vivli’s datasets provide a rich resource for exploring disease progression, treatment efficacy, and biomarker discovery. The upcoming challenge with the AD Data Initiative aims to attract multidisciplinary teams, encouraging the application of advanced analytical techniques such as AI and machine learning to uncover new insights in AD.

**Conclusion:**

Vivli's platform is uniquely positioned to enhance Alzheimer's disease research by enabling data access and fostering a collaborative research environment. The planned data challenge with the AD Data Initiative represents a unique opportunity to harness the collective expertise of the global research community and accelerate progress in AD. By enhancing dataset discovery interoperability and promoting FAIR data‐sharing principles, Vivli continues to drive meaningful advancements in the field, ultimately contributing to improved patient outcomes and therapeutic innovation.

